# Postoperative Complications in Living Donors for Lung Transplantation

**DOI:** 10.1097/TXD.0000000000001617

**Published:** 2024-04-09

**Authors:** Shin Tanaka, Kento Fujii, Megumi Ishihara, Haruki Choshi, Kei Matsubara, Kohei Hashimoto, Shuji Okahara, Kazuhiko Shien, Ken Suzawa, Kentaroh Miyoshi, Hiromasa Yamamoto, Mikio Okazaki, Seiichiro Sugimoto, Shinichi Toyooka

**Affiliations:** 1 Department of General Thoracic Surgery and Breast and Endocrinological Surgery, Okayama University Graduate School of Medicine, Dentistry and Pharmaceutical Sciences, Okayama, Japan.; 2 Department of General Thoracic Surgery and Organ Transplant Center, Okayama University Hospital, Okayama, Japan.; 3 Department of Anesthesiology and Resuscitology, Graduate School of Medicine, Dentistry, and Pharmaceutical Sciences, Okayama University, Okayama, Japan.

## Abstract

**Background.:**

Living donor lobar lung transplantation is a life-saving procedure for critically ill patients. This requires 2 healthy donors exposed to risks and without medical benefit. Therefore, the donor’s safety and minimal postoperative complications are crucial. This study aimed to investigate the short-term outcomes and identify the risk factors affecting these outcomes.

**Methods.:**

The data of 175 living donors enrolled between 1998 and 2022 were analyzed. Donors were divided into era 1 (1998–2009) and era 2 (2010–2022).

**Results.:**

The overall incidence of postoperative complications was 39%, of which 7% were major complications. Donors who underwent surgery on the right side had a higher incidence of delayed pulmonary fistulae (*P* = 0.01) and elevated liver enzyme levels (*P* = 0.028). Living donor surgery on the right side (*P* = 0.01), era 2 (*P* = 0.01), and the need for plasty (*P* = 0.04) were predictors of postoperative complications.

**Conclusions.:**

Updated data on complications and their correlation with postoperative quality of life from this study could aid in the selection of potential donors and facilitate informed consent.

Living donor lobar lung transplantation (LDLLT) is an established life-saving therapy for critically ill patients who cannot wait for deceased donor lung transplantation owing to a donor organ shortage. In LDLLT, 2 healthy donors are usually required for each recipient and are exposed to risks of complications and mortality resulting from the donor operation. Living donors do not medically benefit from this procedure. Therefore, LDLLT can only be performed if the donor’s safety during surgery is assured and if minimal postoperative complications of the procedure are guaranteed.

Although several studies have described the preoperative and postoperative characteristics and assessed the short-term outcomes of living lobar lung donors, no study has been conducted since the last report in 2015.^1^ The postoperative complications rate is unique because the living lung donation operation is more invasive and differs from standard lobectomy, typically performed for malignancy. This procedure requires extensive peritracheal and perivascular dissection, pericardiotomy, and careful fissure dissection to adequately remove the cuff of the bronchus, pulmonary artery, and vein within the lung lobe for successful donor lobe implantation into the recipient. Accurate, reliable, and comprehensive outcome data on living lung donors will provide potential donors with reliable information for donor evaluation and informed consent decisions regarding surgery. This study aimed to investigate the short-term outcomes and identify the risk factors affecting these outcomes of living donors’ lung transplantations at a high-volume center in Japan.

## MATERIALS AND METHODS

### Patients

We invited and studied 175 donors who underwent lung lobectomy at our institution between January 1998, when the first LDLLT was performed in Japan, and December 2022. A lung transplant database containing the characteristics and outcomes of the donors was retrospectively analyzed. The study protocol (1905-022) was approved on June 25, 2021, by the Institutional Review Board of Okayama University Hospital and was conducted in accordance with the Declaration of Helsinki.

### Donor Selection

Our hospital only accepts third-degree blood relatives or a spouse as living donors. The inclusion criteria were healthy individuals aged between 20 and 60 y who provided informed consent; had no infectious diseases, no present or past malignant disease, no abnormalities on electrocardiographs and echocardiograms, no significant medical history or active medical problems, no significant pulmonary pathology on computed tomography on the donor side, and no previous thoracic operation on the side to be donated; and were nonsmokers (if current smokers, smoking cessation is required at the time of the offer for donation and continuous cessation is required after donor lobectomy). For donor safety, the donor’s forced vital capacity (FVC) and forced expiratory volume in 1 s should be >85% of the predicted value. Furthermore, for the donor’s FVC, the adaptation criteria vary depending on the FVC of the recipient and other donors to provide adequate lung capacity for the recipient. Regarding the size-matching protocol, because the right lower lobe consists of 5 segments, the left lower lobe of 4, and the whole lung of 19, the total FVC of the 2 grafts was estimated using the following equation: total FVC of the 2 grafts = measured FVC of the right-side donor × 5/19 + measured FVC of the left-side donor × 4/19. When the total FVC of the 2 grafts was >50% of the predicted FVC of the recipient, we accepted the size discrepancy, irrespective of the recipient’s diagnosis.

### Clinical Data

The enrolled donors’ clinical data include the date of surgery, age, sex, body mass index, type of graft (right lower lobe, left lower lobe, right segment, or left segment), operative time, blood loss, postoperative complications (minor, major, mortality, delayed pulmonary fistula, pneumothorax after chest tube removal, pleuritic, chylothorax, reaccumulation of pleural effusion, bronchial stenosis, empyema, elevated liver enzyme, bleeding from the lung parenchyma, incisional surgical site infection, incision opening, neuralgia, or reoperation), postoperative length of stay, and readmission within 1 mo after discharge. The study period was divided into 2 periods (eras): era 1 (1998–2009) and era 2 (2010–2022). This division followed the 2010 revision of Japan’s transplant law, which led to a gradual increase in deceased donors and a more careful selection of living donors. Additionally, programmatic changes were implemented at our institution, including the adoption of a thoracoscopic approach during surgical procedures. Postoperative complications were evaluated using the Clavien-Dindo classification and classified as minor (grades 1–2) or major (grades 3–4) complications.^2^ Minor complications included deviations from a normal postoperative course and morbidities requiring treatment with drugs, transfusions, and total parenteral nutrition. Major complications included morbidities requiring surgical, endoscopic, or radiological interventions (with or without general anesthesia) and life-threatening complications. Postoperative complications within 3 mo were defined as short-term outcomes.

### Surgical Technique

An epidural catheter was placed in the donor 1 d before the surgery because intraoperative heparinization was planned. Donor lobectomies were performed following a routine procedure described previously.^3,4^ Posterolateral thoracotomy was performed through the fifth intercostal space. If the branches of the middle lobe and lingular arteries were small, they were severed by ligation and division. Ten thousand units of heparin and 500 mg of methylprednisolone were administered intravenously just before preparation for the pneumonectomy for the recipient surgery. After placing the vascular clamps in the appropriate positions, the pulmonary vein, pulmonary artery, and bronchus were divided. The lower lobes were extracted from the donors. The pulmonary vein stumps were oversewn with 5-0 prolene (Ethicon Inc, Tokyo, Japan), and pulmonary artery stumps were oversewn with 6-0 prolene. The bronchial stump was closed using 4-0 PDS interrupted sutures and covered with pericardial fat tissue. Heparinization was reversed by protamine administration. The thoracotomy was closed in the standard manner after chest tube placement.

### Postoperative Management

A standardized multimodal analgesic protocol was administered, including epidural analgesia, nonsteroidal anti-inflammatory drugs, and acetaminophen. The patients were managed in an intensive care unit for the first 24 h and transferred to a regular surgical ward the day after the surgery. The ward nursing staff initiated postoperative ambulation on postoperative day 1. Antithrombotic prophylaxis was not routinely administered owing to the risk of epidural hematoma. The chest tube was removed when air leaks were absent and the fluid output was <200 mL/24 h. A few days before the patient was discharged, a routine bronchoscopy was performed to confirm bronchial patency and sutures’ tendency to heal. The donors attended their first outpatient clinic visit 2 wk after discharge and were followed up at 1 and 3 mo postoperatively. Only 7 institutions in Japan perform LDLLT; most donors are located far away from our institution, and most cases are completed within 3 mo of postoperative follow-up for healthy donors if they have no special requests.

### Statistical Analysis

The overall donor characteristics and outcomes were evaluated, and outcomes based on different era groups and graft types were analyzed. Continuous variables are presented as medians with interquartile ranges (IQRs). Differences between the groups were assessed using the Fisher exact test or chi-square test for categorical variables and the Mann-Whitney *U* test for continuous variables. Multivariate logistic regression analysis was performed to identify the significant risk factors associated with postoperative complications. Statistical significance was set at a *P* value of <0.05. JMP version 16 (SAS Institute Inc, Cary, NC) was used for all statistical analyses.

## RESULTS

### Study Cohort

The demographic characteristics of the 175 donors are shown in Table 1. The donor population consisted of 71 men and 104 women, with a median age of 38 y (IQR, 33–47 y). As shown in Table 2, 90 (51%) were right lobe grafts, 82 (47%) were left lobe grafts, 2 (1%) were right segment grafts, and 1 (1%) was a left segment graft. The median operative time and blood loss were 5.2 h (IQR, 4.5–6.2) and 185 mL (IQR, 108–270), respectively. The overall incidence of postoperative complications was 39%, of which 32% were minor and 6.9% were major complications (Table 3). One patient underwent reoperation because of postoperative bleeding; however, no mortality was recorded. The median postoperative hospital stay was 13 d (IQR, 11–15). The readmission rate within 1 mo of discharge was 1% owing to pleuritis (n = 1) or empyema (n = 1).

**TABLE 1.** Donor characteristics between 1998 and 2022

Variables	Total (N = 175)	Era 1 (1998–2009) (N = 104)	Era 2 (2010–2022) (N = 71)	*P*
Age, y	38 (33–47)	39 (39–49)	38 (34–43)	0.99
Sex				0.65
Male	71 (40%)	55 (53%)	35 (49%)	
Female	104 (59%)	49 (47%)	36 (51%)	
Body mass index, kg/m^2^	22.1 (20.4–24.9)	21.9 (20.6–24.8)	22.4 (20.0–25.1)	0.76
Relations to the recipient				0.0002
Child	27 (15%)	14 (13%)	13 (18%)	
Parent	77 (44%)	36 (35%)	41 (58%)	
Sibling	49 (28%)	40 (38%)	9 (13%)	
Spouse	18 (11%)	14 (13%)	4 (6%)	
Aunt/uncle	3 (2%)	0 (0%)	3 (4%)	
Grandparent	1 (1%)	0 (0%)	1 (1%)	
FVC-based size matching, %	109 (100–118)	111 (104–129)	104 (96–117)	0.004
FEV_1_, %	101 (92.7–111)	102 (95.7–112)	97.5 (90–111)	0.047
Smoking history				0.65
Yes	80 (46%)	49 (47%)	31 (44%)	
No	95 (54%)	55 (53%)	40 (56%)	
Comorbidity				0.66
Yes	27 (15%)	15 (14%)	12 (17%)	
No	148 (88%)	89 (86%)	59 (83%)	
PaO_2_/FiO_2_ ratio	467 (436–503)	464 (435–500)	469 (443–506)	0.34

Data are presented as median (range) or n (%).

FEV_1_, forced expiratory volume in 1 s; FVC, forced vital capacity; PaO_2_/FiO_2_, partial oxygen pressure over fractional inspired oxygen concentration.

**TABLE 2.** Operative findings in living donors between 1998 and 2022

Variables	Total (N = 175)	Era 1 (1998–2009) (N = 104)	Era 2 (2010–2022) (N = 71)	*P*
Surgical procedures				0.21
Right lower lobectomy	90 (51%)	54 (52%)	36 (51%)	
Left lower lobectomy	82 (47%)	50 (48%)	32 (45%)	
Right segmentectomy	2 (1%)	0 (0%)	2 (3%)	
Left segmentectomy	1 (1%)	0 (0%)	1 (1%)	
Plasty				0.05
Pulmonary artery	26 (15%)	16 (15%)	10 (14%)	
Pulmonary vein	2 (1%)	0 (0%)	2 (3%)	
Bronchus	11 (6%)	10 (10%)	1 (1%)	
No	136 (78%)	78 (75%)	58 (82%)	
Operative time, h	5.2 (4.5–6.2)	5.0 (4.3–5.6)	5.6 (4.7–6.8)	0.0006
Blood loss, mL	185 (108–270)	220 (150–300)	120 (50–195)	<0.0001

Data are presented as median (range) or n (%).

**TABLE 3.** Early postoperative outcomes in living donors between 1998 and 2022

Variables	Total (N = 175)	Era 1 (1998–2009) (N = 104)	Era 2 (2010–2022) (N = 71)	*P*
Postoperative complications before discharge	68 (39%)	33 (32%)	35 (49%)	0.0192
Minor complications (CDc 1–2)	56 (32%)	25 (24%)	31 (43%)	0.0063
Major complications (CDc 3–4)	12 (7%)	8 (8%)	4 (6%)	0.6
Delayed pulmonary fistula	7 (4%)	4 (4%)	3 (4%)	0.9
Pneumothorax after chest tube removal	4 (2%)	1 (1%)	3 (4%)	0.16
Pleuritis	4 (2%)	0 (0%)	4 (6%)	0.0067
Chylothorax	1 (1%)	1 (1%)	0 (0%)	0.4
Reaccumulation of pleural effusion	5 (3%)	2 (2%)	3 (4%)	0.37
Bronchial stenosis	3 (2%)	1 (1%)	2 (3%)	0.35
Empyema	1 (1%)	1 (1%)	0 (0%)	0.4
Elevated liver enzyme	23 (13%)	13 (13%)	10 (14%)	0.76
Bleeding from the lung parenchyma	3 (2%)	2 (2%)	1 (1%)	0.8
Incisional surgical site infection	5 (3%)	2 (2%)	3 (4%)	0.37
Incision opening	4 (2%)	2 (2%)	2 (3%)	0.7
Neuralgia	6 (3%)	2 (2%)	4 (6%)	0.19
Reoperation	1 (1%)	1 (1%)	0 (0%)	0.4
Hospital stay, d	13 (11–15)	13 (11–15)	12 (10–15)	0.16
Readmission within 1 mo	2 (1%)	2 (2%)	0 (0%)	0.24

Data are presented as median (range) or n (%).

CDc, Clavien-Dindo classification.

### Effects of the Era (Intervention Period) on Outcomes

**FIGURE 1.**
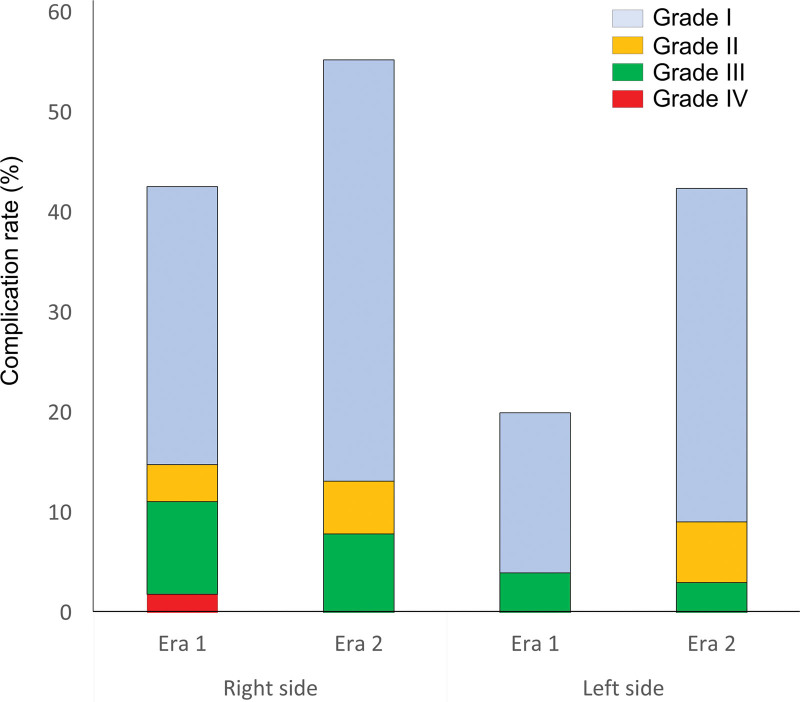
The incidences of postoperative complications of each era in each graft type.

Postoperative outcomes are shown in Tables 1–3. Of the 175 donors, there were 104 cases in era 1 (1998–2009) and 71 cases in era 2 (2010–2022). A significant difference in the recipient was found between the different eras. More than half of the donors were the parents of recipients in era 2. The blood loss slightly decreased from era 1 to era 2. Concerning postoperative complications, the incidence of minor complications tended to increase from era 2 to era 1 (43% and 24%, respectively; *P *= 0.006); however, the incidence of major complications was similar between the 2 eras (8% versus 6%; *P* = 0.6; Figure 1). Individual complications did not differ significantly between the eras, except for pleuritis.

### Effects of the Graft Type on Outcomes

The characteristics and outcomes of the different graft types are summarized in Tables 4 and 5, respectively. Male donors were more likely to undergo right-sided surgeries (68%) than left-sided surgeries (33%). Right-sided operation was associated with a high rate of delayed pulmonary fistula (8% versus 0%, *P* = 0.01) and elevated liver enzyme levels (19% versus 7%, *P* = 0.028) after the operation. The frequency of postoperative complications was higher in the right-sided operation group; however, the incidence of minor and major postoperative complications did not significantly differ between the 2 groups (38% versus 25%, *P* = 0.07 and 10% versus 4%, *P* = 0.11, respectively).

**TABLE 4.** Donor characteristics based on the surgical procedure

Variables	Right side (N = 92)	Left side (N = 83)	*P*
Age, y	38 (29–43)	40 (34–50)	0.0079
Sex			<0.0001
Male	63 (68%)	27 (33%)	
Female	29 (32%)	56 (67%)	
Body mass index, kg/m^2^	22.6 (20.8–26.1)	21.6 (20.1–24.5)	0.036
Relations to the recipient			0.83
Child	16 (17%)	11 (13%)	
Parent	40 (43%)	37 (45%)	
Sibling	25 (27%)	24 (29%)	
Spouse	10 (11%)	8 (10%)	
Aunt/uncle	1 (1%)	2 (2%)	
Grandparent	0 (%)	1 (1%)	
FVC-based size matching, %	110 (103–134)	107 (100–118)	0.29
FEV_1_, %	101 (93–116)	101 (91–113)	0.85
Smoking history			0.034
Yes	49 (53%)	31 (37%)	
No	43 (47%)	52 (63%)	
Comorbidity			0.94
Yes	14 (15%)	13 (16%)	
No	78 (85%)	70 (84%)	
PaO_2_/FiO_2_ ratio	466 (436–504)	468 (437–503)	0.85

Data are presented as median (range) or n (%).

FEV_1_, forced expiratory volume in 1 s; FVC, forced vital capacity; PaO_2_/FiO_2_, partial oxygen pressure over fractional inspired oxygen concentration.

**TABLE 5.** Early outcomes based on the surgical procedure

Variables	Right lower lobectomy (N = 92)	Left lower lobectomy (N = 83)	*P*
Operative time, min	5.2 (4.3–6.3)	5.1 (4.6–5.9)	0.79
Blood loss, mL	200 (113–280)	160 (100–255)	0.1
Postoperative complications before discharge	44 (41%)	24 (29%)	0.001
Minor complications (CDc 1–2)	35 (38%)	21 (25%)	0.07
Major complications (CDc 3–4)	9 (10%)	3 (4%)	0.11
Delayed pulmonary fistula	7 (8%)	0 (0%)	0.01
Pneumothorax after chest tube removal	2 (2%)	2 (2%)	0.91
Pleuritis	1 (1%)	3 (4%)	0.26
Chylothorax	1 (1%)	0 (0%)	0.34
Reaccumulation of pleural effusion	3 (3%)	2 (2%)	0.74
Bronchial stenosis	2 (2%)	1 (1%)	0.62
Empyema	0 (0%)	1 (1%)	0.29
Elevated liver enzyme	17 (19%)	6 (7%)	0.028
Bleeding from the lung parenchyma	3 (3%)	0 (0%)	0.1
Incisional surgical site infection	1 (1%)	4 (5%)	0.14
Incision opening	3 (3%)	1 (1%)	0.36
Neuralgia	2 (2%)	4 (5%)	0.34
Reoperation	1 (1%)	0 (0%)	0.34
Hospital stay, d	13 (11–15)	12 (10–14)	0.07
Readmission within 1 mo	1 (1%)	1 (1%)	0.94

Data are presented as median (range) or n (%).

CDc, Clavien-Dindo classification.

### Predictors for Postoperative Complications

Multivariate logistic regression for any postoperative complications showed a significantly increased risk of living donor surgery on the right side (odds ratio 2.35, *P* = 0.01), in era 2 (odds ratio 2.43, *P* = 0.01), and with the need for plasty (odds ratio 2.25, *P* = 0.04; Table 6). The same analysis was performed for major postoperative complications, but no risk factors were revealed (Table 7).

**TABLE 6.** Multivariable logistic regression analysis for complication (CDc1–4)

Covariates	Estimate (95% CI)	*P*
Side (right)	2.35 (1.21-4.56)	0.01
Era (2010~)	2.43 (1.26-4.71)	0.01
Plasty	2.25 (1.03-4.91)	0.04
Smoking history	1.68 (0.87-3.21)	0.12
Comorbidity	0.93 (0.38-2.29)	0.87

CDc, Clavien-Dindo classification; CI, confidence interval.

**TABLE 7.** Multivariable logistic regression analysis for major complication (CDc3–4)

Covariates	Estimate (95% CI)	*P*
Side (right)	2.59 (0.66-10.2)	0.15
Era (2010~)	0.79 (0.22-2.82)	0.71
Plasty	1.17 (0.29-4.76)	0.83
Smoking history	2.23 (0.63-7.92)	0.20
Comorbidity	0.45 (0.05-3.78)	0.42

CDc, Clavien-Dindo classification; CI, confidence interval.

## DISCUSSION

This large single-center study investigated the short-term outcomes of 175 living donors for lung transplantation in a high-volume center in Japan. The overall incidence of postoperative complications was 39%. The rate of major postoperative complications after the donor operation was 6.9%, and the outcomes based on preoperative variables did not differ significantly. The complication rate is significantly higher than that observed in lower lobectomy for diseases such as lung cancer. This may be attributed to the necessity of suture lines for anastomosis of the transplant, which renders staples unusable at the ends and necessitates frequent plasty. These findings are similar to those of other studies.^1,5,6^ The most common minor complication is elevated liver enzymes (13%), possibly due to the stress of surgery, anesthesia, and heavy use of acetaminophen for postoperative pain.

The Japan Organ Transplant Law was amended in 2010, and the number of deceased donors has gradually increased, resulting in a decrease in living donor lung transplants. Children with fewer deceased donors are more likely to be eligible for living donor transplants, and the proportion of parents as donors was inevitably higher in era 2 (58%) than in era 1 (35%; Table 1). In transplantations for younger pediatric recipients (aged approximately 7 y), nonstandard procedures of middle-lobe lung transplant and segmental lung transplant have been reported since the adult lower lobe is too large for such patients (Table 2).^7,8^ The higher incidence of minor postoperative complications in era 2 than in era 1 (43% versus 24%, *P* = 0.063; Table 3) might be due to the approximately 30% reduction in the number of LDLLT in era 2, making it harder for the era1 medical team to pass on their experience and expertise in perioperative management to era2 medical team. However, in era 2, intraoperative blood loss decreased (Table 2), there were no postoperative readmissions, and the rates of major complications were comparable (Figure 1).

Because male individuals tend to have larger lung volumes than female individuals, male donors are frequently selected for right lower lobe grafts with larger lung volumes (Table 4). Furthermore, liver enzyme levels were higher in the right-side donor because surgical and anesthesia stress were higher on the right side, and both operative time and blood loss were higher on the right side. There were more postoperative complications on the right side than on the left side, which was attributed to a delayed pulmonary fistula (Table 5). Incomplete or absent oblique fissures can be observed more often in the right lung than in the left lung.^9,10^ In living lung transplantation surgery, a stapler is not used whenever possible during anatomical resection to prevent volume loss in the transplanted donor grafts. This may result in a higher risk of delayed pulmonary fistula in right lung donors with increased segmental lobe failure.^11^

Multivariate analysis of postoperative complication (CDc1–4) showed that the presence of plasty was also a risk factor, in addition to the period and the side of the donor operation (Table 6). Plasty surgery was performed in 39 cases to ensure adequate margins for suturing to the recipient’s side, which is a characteristic procedure for donor surgeries. Of the 39 cases, major complications included pleural effusion requiring drainage in 3, pneumothorax in 3, hemorrhage in 2, bronchial stenosis in 2, and pyothorax in 1. However, in multivariate analysis, plasty was not a risk factors for major complications (CDc3–4; Table 7).

LDLLT remains a valuable life-saving option for patients with severe respiratory disorders in Japan. No emergency lung allocation system exists, and the number of deceased pediatric donors is low. Therefore, it is important to develop techniques to ensure the safety of living donor surgeries.

While searching for postoperative complications in the donors, we found 1 donor who developed interstitial pneumonia 3 y after surgery. The recipient had undergone living donor lung transplantation for idiopathic interstitial pneumonia; however, because her mother had interstitial pneumonia, genetic screening was performed, and familial origin was ruled out. According to a precise genetic test performed after lung transplantation, the patient had coatomer protein subunit alpha syndrome, an inherited autosomal dominant disease first reported in 2015.^12,13^ We could not exclude this condition preoperatively because it is a rare form of familial interstitial pneumonia. A careful family history is necessary, and if heredity is suspected, further genetic testing should be performed.^14^

There are several limitations inherent to this study design. First, it was retrospective and investigated a patient cohort during a long period (from 1998 to 2022). Our lung preservation protocol, methods, and management of recipients have changed to a certain extent over time. Second, the study was relatively small scale and was conducted at a single center. However, we believe that this study successfully examined postoperative donor outcomes by collecting extensively detailed donor and objective data. Finally, this study did not demonstrate recipient outcomes because it focused only on donor outcomes. However, no impact of graft selection on recipient outcomes in living donor lung transplantation has been reported under the proper selection criteria for donors and recipients.^15^

In conclusion, living donor lobectomies have been safely performed at our institution with low morbidity and without mortality since the first living lung transplantation in Japan. The detailed and updated results of this study may contribute to donor selection and informed consent requirements.

## ACKNOWLEDGMENTS

The authors thank all staff from the departments involved in our Lung Transplant Program: Respiratory Medicine, Anesthesiology, Intensive Care Unit Pathology, Bacteriology, Rehabilitation Medicine, and acknowledge all the local coordinators for the support provided to the recipients and donors.

## References

[1] Chen F Yamada T Sato M. Postoperative pulmonary function and complications in living-donor lobectomy. J Heart Lung Transplant. 2015;34:1089–1094. 25940076 10.1016/j.healun.2015.03.016

[2] Clavien PA Barkun J de Oliveira ML. The Clavien-Dindo classification of surgical complications: five-year experience. Ann Surg. 2009;250:187–196. 19638912 10.1097/SLA.0b013e3181b13ca2

[3] Date H Aoe M Nagahiro I. Living-donor lobar lung transplantation for various lung diseases. J Thorac Cardiovasc Surg. 2003;126:476–481. 12928647 10.1016/s0022-5223(03)00235-6

[4] Chen F Miwa S Bando T. Pulmonary arterioplasty for the remaining arterial stump of the donor and the arterial cuff of the donor graft in living-donor lobar lung transplantation. Eur J Cardiothorac Surg. 2012;42:e138–e139. 22898400 10.1093/ejcts/ezs460

[5] Battafarano RJ Anderson RC Meyers BF. Perioperative complications after living donor lobectomy. J Thorac Cardiovasc Surg. 2000;120:909–915. 11044317 10.1067/mtc.2000.110685

[6] Yusen RD Hong BA Messersmith EE; RELIVE Study Group. Morbidity and mortality of live lung donation: results from the RELIVE study. Am J Transplant. 2014;14:1846–1852. 25039865 10.1111/ajt.12771 PMC4152404

[7] Otani S Yamamoto H Tanaka S. Paediatric lung transplantation: the impact of age on the survival. Surg Today. 2022;52:1540–1550. 35357572 10.1007/s00595-022-02492-w

[8] Nakajima D Tanaka S Ikeda T. Living-donor segmental lung transplantation for pediatric patients. J Thorac Cardiovasc Surg. 2022;165:2193–2201. 36088146 10.1016/j.jtcvs.2022.07.031

[9] Bostanci K Ozyurtkan MO Polat MO. Variations in pulmonary fissural anatomy: a medicolegal autopsy study of 256 cases. ANZ J Surg. 2020;90:608–611. 31709740 10.1111/ans.15553

[10] Joshi A Mittal P Rai AM. Variations in pulmonary fissure: a source of collateral ventilation and its clinical significance. Cureus. 2022;14:e23121. 35425671 10.7759/cureus.23121 PMC9004548

[11] Geraci TC Chang SH Shah SK. Postoperative air leaks after lung surgery: predictors, intraoperative techniques, and postoperative management. Thorac Surg Clin. 2021;31:161–169. 33926669 10.1016/j.thorsurg.2021.02.005

[12] Watkin LB Jessen B Wiszniewski W; Baylor-Hopkins Center for Mendelian Genomics. COPA mutations impair ER-Golgi transport and cause hereditary autoimmune-mediated lung disease and arthritis. Nat Genet. 2015;47:654–660. 25894502 10.1038/ng.3279 PMC4513663

[13] Patwardhan A Spencer CH. An unprecedented COPA gene mutation in two patients in the same family: comparative clinical analysis of newly reported patients with other known COPA gene mutations. Pediatr Rheumatol Online J. 2019;17:59. 31455335 10.1186/s12969-019-0359-9 PMC6712851

[14] Yoshiyasu N Sato M Urushiyama H. Familial interstitial pneumonia revealed after living-donor lobar lung transplantation. Ann Thorac Surg. 2021;112:e365–e368. 33662313 10.1016/j.athoracsur.2021.02.021

[15] Sugimoto S Date H Miyoshi K. Long-term outcomes of living-donor lobar lung transplantation. J Thorac Cardiovasc Surg. 2022;164:440–448. 34895720 10.1016/j.jtcvs.2021.08.090

